# Integration of HIV Testing into Maternal, Newborn, and Child Health Weeks for Improved Case Finding and Linkage to Prevention of Mother-to-Child Transmission Services in Benue State, Nigeria

**DOI:** 10.3389/fpubh.2017.00071

**Published:** 2017-04-10

**Authors:** Olusoji Akinleye, Gideon Dura, Arjan de Wagt, Abiola Davies, Dick Chamla

**Affiliations:** ^1^UNICEF Field Office, Enugu, Benue State, Nigeria; ^2^Benue State AIDS Control Agency, Ministry of Health, Makurdi, Nigeria; ^3^HIV Program, UNICEF, Abuja, Nigeria; ^4^Emergency Response Team (ERT) Health Section, UNICEF, New York, NY, USA

**Keywords:** integration, HIV testing, maternal, newborn, and child health weeks, antenatal care, prevention of mother-to-child transmission

## Abstract

**Background:**

In Nigeria, maternal, newborn, and child health (MNCH) weeks are campaign-like events designed to accelerate progress toward Millennium Development Goals. The authors examined whether integrating HIV testing into MNCH weeks was feasible and could lead to increased case finding and linkage to prevention of mother-to-child transmission (PMTCT) services.

**Methods:**

Pregnant women attending MNCH week during the first week of December 2014 in 13 local government areas in Benue State were provided with HIV tests and referrals to PMTCT services. Demographic, past antenatal care (ANC), and HIV testing information were collected using a structured questionnaire. We used routine ANC/PMTCT data from national electronic system (DHIS-2) to compare with the results obtained from MNCH week.

**Results:**

A total of 50,271 pregnant women with a median age of 25 years (IQR: 21–29) were offered HIV testing. About 50,253 (99.96%) agreed to get HIV testing, with 1,063 (2.1%) testing positive. Six hundred forty-four (60.6%) of those with positive results were linked to PMTCT. In multivariate analysis, marital status, gestation age, and those with no ANC visit during this pregnancy were associated with a positive HIV test. Approximately 30% (50,253 versus 39,080) more pregnant women received HIV testing in MNCH week compared to those who received HIV testing in routine ANC services in 2013. Of the 50,253 who accepted testing, 15,611 (31.1%) did not attend ANC during this pregnancy, of which 9,615 (61.6%) had not had any previous HIV tests. Four hundred forty-two (4.6%) of these 9,615 tested HIV-positive.

**Conclusion:**

Integration of HIV testing into MNCH weeks is feasible and improved uptake of HIV testing and linkage to care. However, the rate of HIV positivity was lower than that reported by previous studies. The findings indicate that MNCH weeks provides opportunity to reach those who do not attend ANC services for HIV care.

## Introduction

Nigeria accounts for 9% of the global population of people living with HIV, second only to South Africa (1, 2). The country harbors 10% of new HIV infections and 30% of children living with HIV globally (2). This is likely due to low coverage of prevention of mother-to-child transmission (PMTCT) services measuring less than 28% (2). This presented a barrier to achieving the goal of reducing mother-to-child transmission of HIV (eMTCT) to less than 5% MTCT by 2015 (1–3). Limited access to antenatal care (ANC) services is one of the reasons cited for the low coverage of PMTCT (4). In the last Nigerian National HIV/AIDS and Reproductive Health Survey, only 65% of respondents reported attending ANC during their pregnancies in the last 5 years (5). The survey also showed that 48.1% received HIV testing during the last or current pregnancy with the HIV prevalence of 6%. In addition, limited access to PMTCT services due to geographical reasons, social and financial barriers have also been reported (6, 7). As of 2012, only 5% of an estimated 26,000 health facilities in the country offered PMTCT (6). Fear of HIV testing, lack of knowledge of HIV status, confidentiality, and stigma have been documented (7, 8). Nigeria also has the largest gap in HIV testing, particularly among adolescents in western Africa (9).

In order to close the gaps in PMTCT coverage, the Nigeria Presidential Comprehensive Response Plan for HIV/AIDS was launched in 2013. This ambitious plan aimed to increase enrollment of eligible adults and children on ART by 600,000 and activate 2,000 new PMTCT and ART service delivery points across the country. Additionally, a new national eMTCT operational plan identified 14 priority areas of interventions, including ensuring PMTCT be integrated with maternal, newborn, and child health (MNCH) services (10). Achieving these targets required an innovative approach to identify other health service entry points that are in contact with large numbers of pregnant or childbearing women.

Maternal, newborn, and child health weeks are biannual 1-week campaign-like events designed to complement routine ANC services aimed at increasing access to MNCH services. The MNCH weeks were established by the National Council of Health in 2009 to accelerate progress toward Millennium Development Goals (MDGs) number 4 and 5 (11). High attendance by pregnant women and children were observed in MNCH weeks since inception, yet HIV services were not provided—missing a clear opportunity for scaling up HIV testing and linkage to PMTCT services.

Integrated delivery of HIV with other maternal and child health services has been on the rise (12–16); however, few integrated services have been able to measure or document their outcomes following integration. The potential benefits of improved outcomes and efficiency gains associated with integration are encouraging; however, the risk of stigma and increased workloads on health-care workers mean that any integration requires careful design and monitoring (17–19). This report examined the feasibility and acceptability of integrating HIV testing in the MNCH weeks in Benue State, a state with the highest ANC HIV prevalence based on 2010 sentinel surveillance (20). The HIV prevalence and linkage to PMTCT services among pregnant women attending MNCH weeks were also determined, with the aim of generating evidence for an alternative model of service delivery that will improve case finding and linkage to PMTCT services in Nigeria.

## Materials and Methods

This was a cross-sectional study that interviewed and offered HIV testing for all pregnant women attending the MNCH week from 1st to 7th December 2014 in Benue State, Nigeria. During the MNCH week, the services, which included vaccinations for children aged below 5 years and pregnant mothers, iron/folate, malaria bed nets, information on family planning, and hygiene promotion, were provided at health facilities and outreach sites. The eligibility criteria for this study included all pregnant women residing in Benue State receiving any MNCH service during the MNCH week, irrespective of age, socioeconomic or marital status, or gestational age.

The inclusion of free HIV testing and referral to PMTCT into the package of MNCH services if feasible and acceptable, was expected to increase the uptake of PMTCT services by breaking the barriers of accessibility and financial burden that had been reported to limit uptake of services (7). Similarly, it was assumed that the community mobilization preceding the MNCH week could increase awareness and demand for MNCH, including PMTCT services, while overcoming the stigma commonly caused by lack of sufficient knowledge about HIV.

### Study Site and MNCH Week

Benue State is situated in the north central geo-political zone with the highest HIV prevalence among pregnant women attending ANC of 12.7% (20). Inclusion of an HIV test into the package of care was supported by government policy but had not been widely implemented in the past. During the planning of MNCH week, a steering committee was created to oversee the integration of HIV testing and monitor its implementation. This included generating estimate of expected MNCH week’s attendance, resource requirements, and adaptation of the existing guide to ensure HIV testing and linkage to PMTCT services were included in the basic package. In addition, a sub-group of this committee developed a methodology and tools for data collection and for training health workers who were to be involved in HIV testing. There were further subgroups for logistics ensuring HIV test kits and reporting tools were available, and for providing advocacy and community sensitization.

To maximize the participation of pregnant women and HIV testing during the MNCH weeks, community mobilization was conducted. Meetings were held with community leaders discussing the inherent benefits of allowing community members to access services during the week. Messages were also relayed to the general public through religious houses, health facilities, and community announcers. The messages focused on the need and importance of attending MNCH weeks and accepting services provided. These messages were reinforced through radio and television public service announcements. The focus of community sensitization was on overall ANC services and not specifically for HIV testing and/or PMTCT uptake.

### HIV Testing Strategy and Referral to PMTCT

**Figure 1:**
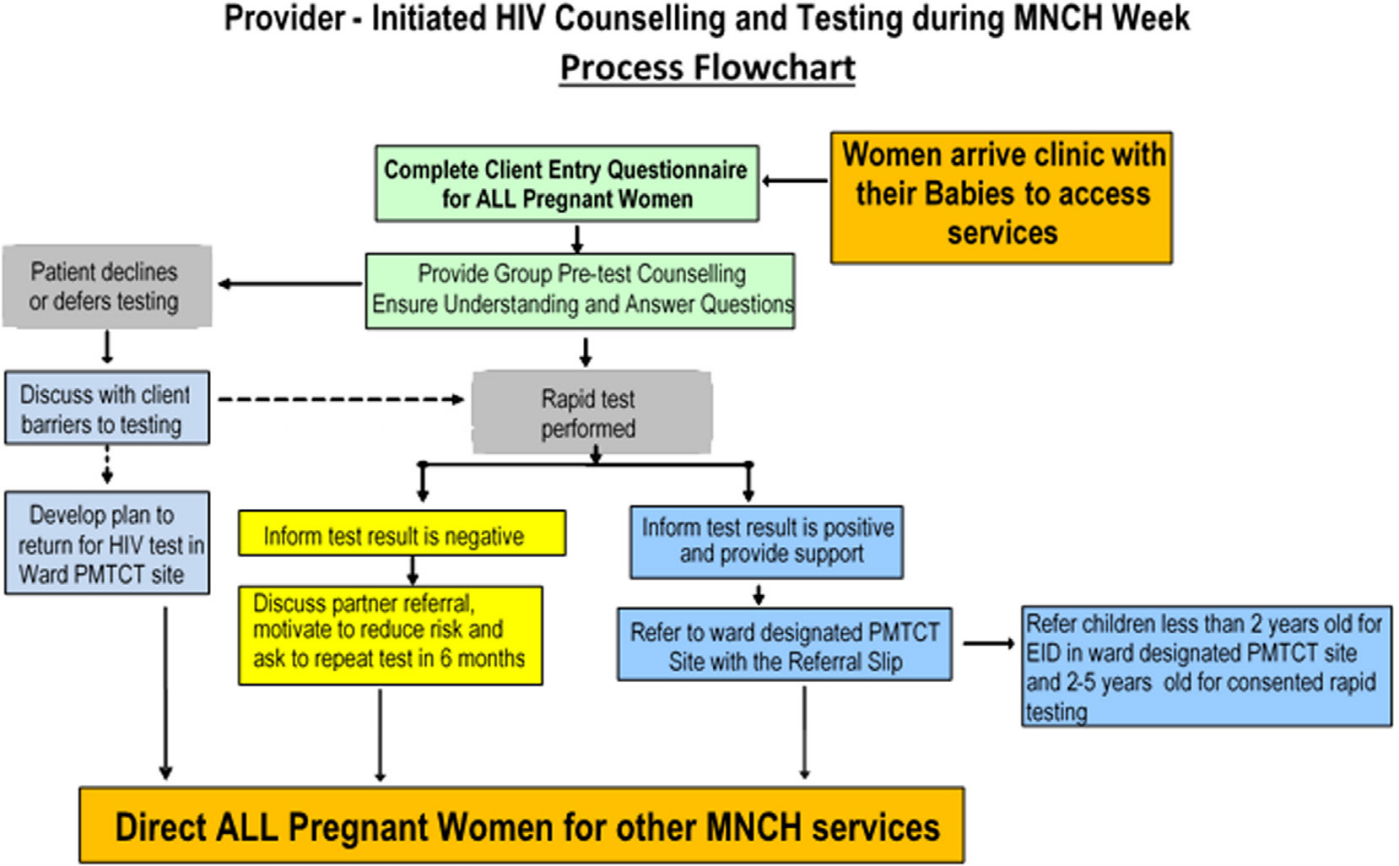
**The client flow during the integrated HIV testing and prevention of mother-to-child transmission (PMTCT) with maternal, newborn, and child health (MNCH) week in Benue State, Nigeria**.

In line with national guidelines, “*Opt-Out*” HIV testing strategy was employed, which did not require signed consent from pregnant women. The HIV testing method started with a pre-test counseling followed by blood collection for those who consented. The Rapid HIV Antibody Test, Alere Determine™ HIV 1/2 Ag/Ab was used and post-test counseling provided to all women irrespective of their HIV test results. Uni-Gold™ HIV (Trinity Biotech) was used as a tiebreaker. The flow of clients for provision of MNCH services and HIV testing is illustrated in Figure 1. For those testing HIV-positive, a “referral slip” was provided for linkage to follow-up their enrollment in PMTCT services.

### Sampling and Data Collection

A total of 13 local government areas (LGAs) and 297 sites were selected and designated as pilot sites for inclusion of HIV into MNCH weeks. It was estimated that the number of pregnant women in the 13 selected LGAs was around 184,686, and that about 50% (90,000) of those would attend MNCH weeks. Based on government statistics, in 2013, the total number of pregnant women receiving HIV testing in routine ANC visits in the 13 LGAs was 39,080 and the coverage of PMTCT was 30%. To statistically detect the 50% acceptance rate with the precision of ±5% at the 95% confidence interval (CI) level, at least 400 pregnant women per each LGA were needed to consent for HIV testing. However, the number of women attending the MNCH weeks and offered HIV testing was higher. The consenting women were consecutively pre-tested, offered HIV testing followed by post-test counseling.

Teams of trained health workers administered a pre-piloted, paper-based questionnaire to collect demographic information, history of ANC attendance, HIV status, and access to PMTCT from pregnant women. Those participants who responded “no” for past HIV testing or PMTCT were also required to provide the main reasons for not being testing or enrolling in PMTCT services. These questions were drawn from past assessment instruments and national PMTCT/ANC registers. Only minimum amount of information was collected in order to reduce the interruptions in the flow and delivery of services during the MNCH weeks. A referral slip, collected at PMTCT referral sites was used to confirm the enrollment of pregnant women who tested HIV-positive. The unique numbers and information from these referral slips were then linked to participant questionnaires. The study supervisors reviewed the completeness of questionnaires daily and in case of missing data, efforts were made to fill missing information using the MNCH week register or tracing the participants using their contact phone numbers provided at MNCH week registration counters. Similarly, the 2013 data from the electronic Nigeria National Response Information Management System (eNNRIMS-DHIS2) and Benue State health authorities, regarding routine ANC attendance and HIV testing, was retrieved in order to compare with data generated during the MNCH week. Data from the questionnaires were entered into a Microsoft Access/excel database, checked for consistency, and uploaded into STATA software for analysis.

### Data Analysis

The primary outcomes were the acceptability of the offer for HIV test, HIV prevalence rate among pregnant women, and proportion of those tested positive who linked to PMTCT services. The acceptability was measured by the proportion of the pregnant women who agreed to test. The HIV prevalence was determined by the proportion of women who had HIV-positive results. New HIV-infected cases were identified by examining the proportion of women who tested positive who had not received HIV testing prior to MNCH weeks. The proportion of women who did not attend ANC during the pregnancy but received HIV testing during MNCH weeks was also determined. Linkage to PMTCT was confirmed by the proportion of women testing positive whose referral slips were submitted and registered at ANC and PMTCT facilities within 1 month post-MNCH week. All responses on the reasons for not receiving HIV testing or PMTCT prior to MNCH weeks were grouped based on recurrent themes.

Bivariate and multivariate analyses were performed to examine the characteristics of pregnant women attending MNCH weeks who accepted a HIV test and factors associated with HIV-positive rates. Statistical association was established using odds ratio (OR) with 95% CI while statistical significance was judged using alpha 0.05. All statistical analyses were completed using STATA version IC 11 (StataCorp LP, TX, USA).

## Results

A total of 50,271 pregnant women with a median age of 25 years (IQR: 21–29) and mean gestational age of 4.7 months (SD: ±2.3) in 13 LGAs were offered HIV testing. Of these, 46,006 (91.5%) were married, 45.5% had completed primary education, and 67.4% were engaged in farming occupations. Approximately 70.3% (35,356/50,271) were aged between 20 and 29 years. The LGAs where the highest number of women was offered tests were Gboko (15%) and Otukpo (14.8%), while lowest number of women offered testing were from Katsina Ala (2.8%). However, all LGAs surpassed the required sample sizes.

### Past History of ANC Attendance and HIV Testing

Approximately 69% (34,660) participants reported at least one ANC visit during the pregnancy and of these, 57.5% were attending government ANC facilities followed by 24. 2 and 10.7% in private and Mission-owned ANC facilities, respectively. Similarly, 35,068 (69.8%) of women reported being tested for HIV in the past, of which 521 (1.5%) reporting being HIV-positive. Of those reporting HIV-positive results, 487 (93.7%) were currently receiving ARVs for PMTCT and 34 (6.3%) were not on any medication for HIV. The most frequent reason given for not receiving HIV testing or PMTCT services prior to this MNCH week was *lack of sufficient money*. This was followed by *I was not aware of the importance, fear of HIV test and positive results, distance to HIV testing sites*, and *stigma and discrimination*. Least cited reasons included *my husband refused, I was not sick*, and *I trust myself and my partner/spouse*.

### Acceptability of HIV Testing, and HIV Prevalence during MNCH Week

The characteristics of pregnant women attending MNCH weeks who accepted HIV testing are presented in Table 1. Of the 50,271 pregnant women, 50,253 (99.96%) accepted HIV testing, with 1,063 (2.1%) showing positive HIV results. This included 290 (0.8%) of 34,547 women who reported being tested HIV-negative in the past. Age, education, marital status, occupation, past ANC visit, or HIV test did not have any association with the acceptability of the HIV testing during the MNCH week (Table 1). The factors that were associated with HIV-positive test results after adjusting for age, marital status, education, and occupation are summarized in Table 2. These factors included gestation age (*p* < 0.05), marital status (*p* < 0.05), and past history of ANC visit (*p* < 0.01). Of the 1,063 who tested HIV-positive, 644 (60.6%) were referred and enrolled to the PMTCT services. In a logistic regression model, women who were enrolled in PMTCT were more likely to be in their first trimester (OR = 1.52; 95% CI: 1.06–2.16, *p* = 0.021), had not attended at least one ANC visit (OR = 0.66; 95% CI: 0.49–0.89, *p* = 0.007), or had not had an HIV test (OR = 0.07; 95% CI: 0.04–0.11, *p* = 0.00) prior to this MNCH week campaign. However, age, education, marital status, or occupation did not influence the linkage to PMTCT services.

**Table 1:** **Characteristics of pregnant women who accepted HIV testing during the maternal, newborn, and child health (MNCH) week, Benue State, Nigeria**.

Characteristics	Accepted HIV testing	Odds ratio (95% confidence interval)	*p* Value
Total number of pregnant women offered HIV testing in Benue State: *n* = 50,271	50,253 (99.9%)		
**Age of women**			
≤19 years (*n* = 6,585)—reference	6,585 (100%)		
20–29 years (*n* = 35,356)	35,353 (99.9%)	8.8 (0.9–84.3)	0.06
30–39 years (*n* = 8,162)	8,147 (99.8%)	0.7 (0.1–5.1)	0.69
40 years and above (*n* = 168)	168 (100%)		
**Gestational age**			
1st trimester (*n* = 18,314)—reference	18,304 (99.9%)		
2nd trimester (*n* = 18,892)	18,885 (99.9%)	1.5 (0.6–3.9)	0.43
3rd trimester (*n* = 13,065)	13,064 (99.9%)	7.1 (0.9–55.8)	0.06
**Education status**			
No formal education (*n* = 11,380)—reference	11,377 (99.9%)		
Primary education (*n* = 22,882)	22,872 (99.9%)	0.6 (0.2–2.2)	0.44
Secondary education (*n* = 14,061)	14,057 (99.9%)	0.9 (0.2–4.1)	0.92
Tertiary education (*n* = 1,948)	1,947 (99.9%)	0.5 (0.1–4.9)	0.56
**Marital status**			
Single (*n* = 2,363)—reference	2,362 (99.9%)		
Married (*n* = 46,006)	45,989 (99.9%)	1.1 (0.2–8.6)	0.90
Separated (*n* = 1,072)	1,072 (100%)		
Divorced (*n* = 290)	290 (100%)		
Widowed (*n* = 540)	540 (100%)		
**Occupation**			
Unemployed (*n* = 4,991)—reference	4,989 (99.9%)		
Farming (*n* = 33,889)	33,880 (99.9%)	1.5 (0.3–7.0)	0.60
Trading (*n* = 9,856)	9,849 (99.9%)	0.6 (0.1–2.7)	0.48
Civil servant (*n* = 1,535)	1,535 (100%)		
**Reported antenatal care (ANC) visit during this pregnancy**			
Yes (*n* = 34,660)	34,645 (99.9%)	0.4 (0.1–1.5)	0.20
No (*n* = 15,611)—reference	15,608 (99.9%)		
**Reported having HIV test in the past**			
Yes (*n* = 35,068)	35,053 (99.9%)	0.5 (0.1–1.6)	0.22
No (*n* = 15,203)—reference	15,200 (99.9%)		
**Reported knowing HIV status before MNCH week**			
Positive (*n* = 521)	520 (99.9%)	0.2 (0.03–1.5)	0.12
Negative (*n* = 34,547)—reference	34,533 (99.9%)		
**Reported currently receiving ARVs for prevention of mother-to-child transmission**			
Yes (*n* = 487)	486 (99.9%)	0.07 (0.004–1.1)	0.13
No (*n* = 34)—reference	34 (100%)		

**Table 2:** **Factors associated with HIV-positive results among pregnant women attending maternal, newborn, and child health weeks in Benue State, Nigeria**.

	HIV-positive test	Odds ratio (OR) [95% confidence interval (95% CI)]	*p* Value	Adj OR (95% CI)	*p* Value
Total number of pregnant women offered HIV testing in Benue State: *n* = 50,271					
**Age of women**					
≤19 years (*n* = 6,585)—reference	90 (1.4%)				
20–29 years (*n* = 35,356)	776 (2.2%)	1.6 (1.3–1.9)	0.00	1.4 (0.9–2.2)	0.11
30–39 years (*n* = 8,162)	192 (2.4%)	1.9 (1.5–2.4)	0.00	1.5 (0.9–2.4)	0.09
40 years and above (*n* = 168)	5 (2.9%)	1.5 (0.9–2.4)	0.06		
**Gestational age**					
1st trimester (*n* = 18,314)—reference	321 (1.8%)				
2nd trimester (*n* = 18,892)	401 (2.1%)	1.2 (1.1–1.4)	0.01	1.2 (0.9–1.6)	0.23
3rd trimester (*n* = 13,065)	341 (2.6%)	1.5 (1.3–1.8)	0.00	1.4 (1.04–1.9)	0.03*
**Education status**					
No formal education (*n* = 11,380)—reference	291 (2.6%)				
Primary education (*n* = 22,882)	493 (2.2%)	0.8 (0.7–0.9)	0.02	1.1 (0.8–1.5)	0.54
Secondary education (*n* = 14,061)	238 (1.7%)	0.7 (0.6–0.8)	0.00	0.9 (0.7–1.4)	0.84
Tertiary education (*n* = 1,948)	41 (2.1%)	0.8 (0.6–1.1)	0.24		
**Marital status**					
Single (*n* = 2,363)—reference	23 (0.97%)				
Married (*n* = 46,006)	971 (2.1%)	2.2 (1.4–3.3)	0.00	3.4 (1.2–9.7)	0.02*
Separated (*n* = 1,072)	22 (2.1%)	2.1 (1.2–3.8)	0.01	2.9 (0.8–11.1)	0.11
Divorced (*n* = 290)	10 (3.5%)	3.6 (1.7–7.7)	0.00	9.8 (2.3–41.2)	0.00*
Widowed (*n* = 540)	37 (6.9%)	7.5 (4.4–12.7)	0.00	4.8 (1.2–18.9)	0.02*
**Occupation**					
Unemployed (*n* = 4,991)—reference	64 (1.3%)				
Farming (*n* = 33,889)	811 (2.4%)	1.9 (1.5–2.4)	0.00	1.3 (0.8–2.1)	0.25
Trading (*n* = 9,856)	155 (1.6%)	1.2 (0.9–1.6)	0.17		
Civil servant (*n* = 1,535)	33 (2.2%)	1.7 (1.1–2.6)	0.02	1.7 (0.8–3.7)	0.21
**Reported ANC visit during this pregnancy**					
Yes (*n* = 34,660)	621 (1.8%)	0.6 (0.5–0.7)	0.00	0.4 (0.3–0.5)	0.00*
No (*n* = 15,611)—reference	442 (2.8%)				
**Reported having HIV test in the past**					
Yes (*n* = 35,068)	776 (2.2%)	1.2 (1.03–1.3)	0.02	1.7 (0.9–3.1)	0.05
No (*n* = 15,203)—reference	287 (1.9%)				

*Denotes those variables with statistical significance.

### Case Finding

Within the 13 selected LGAs, approximately 30% (50,253 versus 39,080) more pregnant women received a HIV test during the MNCH week compared with the total number of pregnant women receiving HIV testing in routine ANC services throughout 2013. The MNCH week also identified 15,611 pregnant women who had not attended any routine ANC visit during the pregnancy. Of these, 61.6% (9,615/15,611) had not been tested for HIV prior to the MNCH week, and 4.6% (442/9,615) tested HIV positive during the MNCH week. In addition, of the 1,063 who tested positive during the MNCH week, only 521 reported positive results prior to the MNCH week, therefore 542 women were newly identified. Similarly, out of 644 cases that were linked to PMTCT, only 69 (10.7%) were currently receiving PMTCT services, therefore 575 (89.3%) were newly enrolled into care.

## Discussion

The results of this study uniquely determine acceptability and outcomes of HIV integration into MNCH weeks among pregnant women. Although HIV prevalence was lower than that reported among women attending routine ANC in the past, these results crucially confirm high acceptability of HIV testing by pregnant women during the MNCH weeks (5, 20, 21), and it should be noted that reports on the prevalence of HIV among pregnant women in Nigeria have been inconsistent. Invariably, the characteristics of pregnant women, HIV testing algorithms, settings, and methodologies between this and the past studies are different, limiting their comparability. It resurrects the long-standing, polarizing debate on utilizing PMTCT program data for HIV surveillance among pregnant women (22, 23). The rate depicted by this paper is, however, closer to the national HIV prevalence rate of 4.1% but lower than that of Benue general population prevalence (5).

The acceptability rate shown by this study is strikingly high and was not influenced by any factor. This indicates that prior knowledge of HIV status, or the risk of stigma and potential lack of confidentiality as reported by other integrated delivery of services (17, 18, 24) did not have significant effect on acceptability. Likewise, the two-third rate of respondents reporting ANC visit during this pregnancy is similar to the rate of ANC attendance reported by others surveys (5, 21).

These findings raise the question as to whether integrating HIV into MNCH weeks is a scalable strategy that can improve case finding and linkage to PMTCT services, ultimately reversing the current low coverage of PMTCT services in Nigeria. The protagonists of this approach might be encouraged by the potential to reach more pregnant women with HIV testing, identify those who have not attended ANC, identify more HIV-positives, and enroll more HIV-positive women to PMTCT services as exhibited by this study. The possibility of information bias related to self-reporting of past ANC attendance and HIV testing should be acknowledged, as well as any bias in quality of routine ANC data that were used to compare with the results of MNCH weeks; however, the findings of this paper further reaffirm the published literature of improved uptake of services following integrated delivery of services (13, 25).

Despite the encouraging results, the findings also raise some critical questions that need further elucidation. The low rate of HIV positivity raises the issue of cost-effectiveness of this strategy. In the past decade, the *value-for-money* ideology has been central to most policy decisions and investment patterns (26, 27). Determining the cost-effectiveness of this strategy, however, requires a comparison intervention. The routine HIV testing among pregnant women attending ANC might be a potential comparator, but to our knowledge data on costing on HIV testing in routine ANC services in Nigeria remains scarce. Similarly, potential negative consequences on the uptake of other MNCH services following their integration have not been examined. The strategy does not address the root causes of low uptake of HIV testing and PMTCT services in the country despite its potential to supplement routine ANC services. These causes of low uptake of HIV testing and PMTCT services have been widely published in Nigeria (6–8, 28–30) and corroborated by the qualitative findings of this study. The health system capacity to cope with potential increases in demands for PMTCT services following HIV integrated MNCH weeks needs careful consideration. Arguably, due to variations in the epidemic context and cultural dimensions among states, there might be questions on whether high acceptability and feasibility of this approach can be replicated across the country.

Despite these arguments, there is a general consensus that can be drawn from the findings of this study. The gap in access to PMTCT services in Nigeria demands novel service delivery models, and HIV integration into MNCH weeks may prove to be one of the effective options. Nigeria national policy recommends HIV testing to all pregnant women, and existing strategies relying on routine HIV testing among ANC attendees, have not been effective in reaching women. Like other PMTCT priority countries, Nigeria strives to accelerate progress toward the elimination of the mother-to-child transmission of HIV and new post-MDG targets (31, 32), in order to achieve these targets, opportunities such as MNCH weeks, should be further optimized to reach more pregnant women. Moving forward, there is a need to reconcile the *value-for-money* and equity idioms. The *high volume, low yield* scenario observed in this initiative mirrors the current dilemmas in countries such as India with high number of pregnant women and low HIV prevalence. Regardless of policy inclination, balancing the costs and expected outcomes warrants the economic evaluations of this initiative in donor-dependent settings such as Nigeria. The costing component should also examine potential increases in new pediatric infections and lives lost in the absence of this initiative. Robust monitoring of bi-directional impacts on both HIV and MNCH outcomes remains critical and will likely promote mutual investments from both programs. Implementation research that addresses unanswered questions should form a part of this initiative during the scale-up phase. Where MNCH weeks are conducted, integrating HIV counseling and testing should be regarded as a supplemental intervention to bolster PMTCT coverage to reach part of the unreached populations.

## Conclusion

Considering continued advances in innovations and simplified antiretroviral therapy, the opportunities for identifying HIV-infected women in MNCH platforms should be optimized for greatest impact. Early identification of HIV-infected pregnant women ensures access to PMTCT services which has far reaching benefits for the health of mothers and their HIV-exposed children. Achieving universal HIV access and other global targets requires multiple and complimentary service delivery models for HIV testing. Integration of HIV into MNCH weeks is one of those complimentary models.

## Ethics Statement

This study and the inclusion of HIV testing into MNCH weeks, as part of the basic care package, was carried out with permission of the federal and Benue State ministries of health in line with Benue State Ethical Committee. Ethical clearance was not required as this was considered by the Benue ministry of health as a project evaluation of integrated delivery of services using programmatic data, conducted in line with national clinical guidelines that recommend HIV testing for all pregnant women, and supervised by the government steering committee (ref: MOH/MED/57/1/52). However, the ministerial approval was required and granted. In addition, personal identifiers such as participants’ names were removed to ensure anonymity and confidentiality. Unique numbers were used instead and linked to participants’ questionnaire and referral slips. All health workers involved in HIV testing and data collection were trained to maintain participants’ confidentiality.

## Author Contributions

Conceived and coordinated data collection and analysis: OA, DC, AD, and GD. Wrote the paper: DC, OA, AW, and AD. Interpretation, editing, and reviewing: DC, OA, AW, AD, and GD.

## Conflict of Interest Statement

The authors declare that the research was conducted in the absence of any commercial or financial relationships that could be construed as a potential conflict of interest.
